# Supplementary material for: Effectiveness of Lifestyle Interventions for Prevention of Harmful Weight Gain among Adolescents from Ethnic Minorities: A Systematic Review
Source: Int J Environ Res Public Health. 2020 Aug 20;17(17):6059. doi: 10.3390/ijerph17176059 (PMC7503574; doi:10.3390/ijerph17176059)
Supplement: Supplementary file 1 [file ijerph-17-06059-s001.zip › 1supplementary ijerph-870986/Table S3 Table S4.pdf]

Table S3 Risk of bias as assessed by the Cochrane Collaboration Tool[32] for the randomized controlled trials included in the systematic review of effectiveness of preventions interventions for adolescents from ethnic/racial minorities (n=7).

First author, year, country, citation	Selection bias									
	Random sequence generation		Allocation concealment		Attrition bias		Detection bias		Reporting bias	
	Cochrane Judgment	Supporting evidence	Cochrane judgment	Supporting evidence	Cochrane judgment	Supporting evidence	Cochrane judgment	Supporting evidence	Cochrane judgment	Supporting evidence
Black *et al.* 2010[33] USA	Unclear risk	Method not stated	Unclear risk	Method not stated	Low risk	ITT analysis performed	Low risk	Research assistants blinded to group allocation and baseline findings.	Unclear risk	Insufficient information
Chen *et al.* 2011[36] USA	Low risk	Computer random number generator	Unclear risk	Method not stated	Low risk	Dropouts balanced across groups (I: 1/27, C: 3/27), reasons for dropping out not stated	Unclear risk	Not reported	Unclear risk	Insufficient information
Ebbeling *et al.* 2006[29] USA	Unclear risk	Method not stated	Low risk	Study statistician randomized group assignments	Low risk	No dropouts	Unclear risk	Not reported	Unclear risk	Insufficient information
Ezendam *et al.* 2012[28] Netherlands	Low risk	Random number generator	Low risk	Randomization was done by independent researcher	Low risk	ITT analysis performed using baseline observation carried forward (BOCF) and	High risk	The researcher and research assistants were not blinded to allocation during data	Low risk	Pre-specified outcomes from protocol reported

First author, year, country, citation	Selection bias				Attrition bias		Detection bias		Reporting bias	
	Random sequence generation		Allocation concealment							
	Cochrane Judgment	Supporting evidence	Cochrane judgment	Supporting evidence	Cochrane judgment	Supporting evidence	Cochrane judgment	Supporting evidence	Cochrane judgment	Supporting evidence
						last observation carried forward (LOCF) procedures		collection and data analyses		
Nollen *et al.* 2012[50], 2014[53], USA	Unclear risk	Method not stated	Unclear risk	Method not stated	Low risk	Nil ITT analysis	Unclear risk	Not reported	Unclear risk	Insufficient information
Singh *et al.* 2007[60], 2009[61] Netherlands	Low risk	SPSS statistical software	Unclear risk	Method not stated	Low risk	ITT analysis performed	High risk	Research assistants not blinded to group allocation	Low risk	Pre-specified outcomes from protocol reported
Whittemore *et al.*2013[68] USA	Unclear risk	Method not stated	Unclear risk	Method not stated	Low risk	ITT analysis performed	Unclear risk	Not reported	Low risk	Pre-specified outcomes from protocol reported

ITT, Intention to Treat;

Table S4 Risk of bias as assessed by the Cochrane Collaboration Tool[32] for the randomized controlled trials included in the systematic review of effectiveness of preventions interventions for adolescents from ethnic/racial minorities (n=23).

**First author, year, country, citation**	**Selection bias**									
	**Random sequence generation**		**Allocation concealment**		**Attrition bias**		**Detection bias**		**Reporting bias**	
	**Cochrane Judgment**	**Supporting evidence**	**Cochrane judgment**	**Supporting evidence**	**Cochrane judgment**	**Supporting evidence**	**Cochrane judgment**	**Supporting evidence**	**Cochrane judgment**	**Supporting evidence**
Amaro *et al*. 2006[32], Italy	Unclear risk	Method not stated	Unclear risk	Method not stated	High risk	Nil ITT analysis	Unclear risk	Not reported	Unclear risk	Insufficient information
Briancon *et al*. 2010[35], France Bonsergent *et al*. 2013[34], France	Unclear risk	Method not stated	Unclear risk	Method not stated	Low risk	ITT analysis performed	Unclear risk	Not reported	Low risk	All pre-specified primary outcomes reported
Dunker *et al*. 2017[38], Brazil	Low risk	Random selection of paper slips	Low risk	Paper slips selected by school principals	Low risk	ITT analysis performed	Low risk	Blinding present during data analysis, (no blinding during assignment and assessment unlikely to have affected outcome)	Low risk	All pre-specified primary outcomes reported
French *et al*. 2011[27], USA	Unclear risk	Method not stated	Unclear risk	Method not stated	Low risk	Dropouts balanced across groups (I: 2/45, C: 1/44), reasons for dropping out not stated	High risk	Assessors and subjects not blinded to group allocation	Unclear risk	Insufficient information

First author, year, country, citation	Selection bias				Attrition bias		Detection bias		Reporting bias	
	Random sequence generation		Allocation concealment							
	Cochrane Judgment	Supporting evidence	Cochrane judgment	Supporting evidence	Cochrane judgment	Supporting evidence	Cochrane judgment	Supporting evidence	Cochrane judgment	Supporting evidence
Haerens *et al*. 2006[40], Belgium	Unclear risk	Method not stated	Unclear risk	Method not stated	High risk	Nil ITT analysis	Unclear risk	Not reported	Unclear risk	Insufficient information
Hollis *et al.* 2016[41], Australia Sutherland *et al.* 2013[65],2016[66,] Australia	Low risk	Computer generated block randomization procedure	Low risk	Allocated by an independent statistician	Low risk	ITT analysis performed	Unclear risk	Study did not address whether blinding occurred	Low risk	Pre-specified outcomes from protocol reported
Leme *et al.* 2015[43], 2016[26], 2018[42] Brazil	Low risk	Paper slips chosen out of a bag	Low risk	Allocated by individual not involved with research project	Low risk	ITT analysis performed	Low risk	Baseline and post-test assessments conducted by trained research assistants blinded to group allocation	Low risk	Pre-specified primary outcomes from protocol reported.
Lindgren *et al*. 2011[44], Sweden	Unclear risk	Method not stated	Unclear risk	Method not stated	High risk	ITT analysis not performed, dropouts not reported (I: 27/54, 21/56) and reasons for dropout not reported	High risk	research team delivered intervention	Unclear risk	Insufficient information
Lubans *et al.* 2011[45], 201,6[48] Australia	Unclear risk	Method not stated	Unclear risk	Method not stated	High risk	ITT analysis not performed, dropouts not	High risk	Research assistants and participants not blinded to allocation	Low risk	Pre-specified outcomes from protocol reported

First author, year, country, citation	Selection bias				Attrition bias		Detection bias		Reporting bias	
	Random sequence generation		Allocation concealment							
	Cochrane Judgment	Supporting evidence	Cochrane judgment	Supporting evidence	Cochrane judgment	Supporting evidence	Cochrane judgment	Supporting evidence	Cochrane judgment	Supporting evidence
						balanced between groups (I: 13/50, C: 5/50) and reasons for dropout not reported				
Lubans *et al.* 2010[46],2012[47] Australia Dewar *et al.* 2013[37], Australia	Unclear risk	Method not stated	Low risk	Allocated by an independent researcher	Low risk	ITT analysis performed	High risk	Research team blinded to group allocation only at baseline	Low risk	Pre-specified outcomes from protocol reported
Melnyk *et al.* 2013[25], USA	Low risk	Placing all of the school names in a hat and then randomly drawing them out	Low risk	Names drawn were assigned either to COPE or control based on being drawn out of the hat.	Unclear risk	Intention to treat? Does not say how. Dropouts not balanced between groups (I: 88/374, C: 93/433) and reasons for dropout not reported	Low risk	Teachers and participants were blinded to study arm. Research assistants collecting measures/outcomes were blinded to study grouping.	Low risk	Pre-specified primary outcomes from protocol reported.
Mihas *et al.* 2009[49], Greece	Low risk	Computer random	Unclear risk	Method not stated	Low risk	Nil ITT analysis	High risk	Research assistants and participants	Unclear risk	Insufficient information

First author, year, country, citation	Selection bias				Attrition bias		Detection bias		Reporting bias	
	Random sequence generation		Allocation concealment							
	Cochrane Judgment	Supporting evidence	Cochrane judgment	Supporting evidence	Cochrane judgment	Supporting evidence	Cochrane judgment	Supporting evidence	Cochrane judgment	Supporting evidence
		number generator						not blinded to allocation		
Neumark *et al.* 2010[24] USA	Unclear risk	Method not stated	Unclear risk	Method not stated	High risk	Nil ITT analysis	High risk	Research staff not blinded	Unclear risk	Insufficient information
Patrick *et al.* 2006[52]	Unclear risk	Method not stated	Unclear risk	Method not stated	Low risk	ITT performed using baseline observation carried forward (BOCF) and last observation carried forward (LOCF) procedures	Unclear risk	Not reported	Unclear risk	Insufficient information
Peralta *et al.* 2009[53] Australia	Low risk	Computer random number generator	Unclear risk	Method not stated	Low risk	ITT analysis performed	Low risk	Measurements conducted by trained independent assessors blind to group allocation	Unclear risk	Insufficient information
Rabiei *et al.* 2017[54], Iran	Unclear risk	Method not stated	Unclear risk	Method not stated	Unclear risk	Dropouts not reported	Unclear risk	Not reported	Unclear risk	Insufficient information
Rodearmel *et al.* 200,7[55] USA	Unclear risk	Method not stated	Unclear risk	Method not stated	High risk	Nil ITT analysis	Unclear risk	Not reported	Unclear risk	Insufficient information

First author, year, country, citation	Selection bias									
	Random sequence generation		Allocation concealment		Attrition bias		Detection bias		Reporting bias	
	Cochrane Judgment	Supporting evidence	Cochrane judgment	Supporting evidence	Cochrane judgment	Supporting evidence	Cochrane judgment	Supporting evidence	Cochrane judgment	Supporting evidence
Simons *et al.* 2014[58], 201,5[57] Netherlands	Low risk	Computer generated block randomization procedure	Unclear risk	Method not stated	Low risk	ITT analysis performed	High Risk	Participants and research assistants blinded at baseline, but not at T4 and T10. Data analyses not blinded	Low risk	Pre-specified outcomes from protocol reported
Singhal *et al.* 2010[62] India	Unclear risk	Method not stated	Unclear risk	Method not stated	Low risk	ITT analysis performed	Unclear risk	Not reported	Unclear risk	Insufficient information
Smith *et al.* 2014[63,64] Australia	Low risk	Computer random number generator	Low risk	Performed by independent researcher	Low risk	ITT analysis performed	Unclear risk	Not reported	Low risk	Pre-specified outcomes from protocol reported
Thakur *et al*. 2016[23], India	Unclear risk	Method not stated	Unclear risk	Method not stated	Low risk	ITT analysis performed	Unclear risk	Not reported	Unclear risk	Insufficient information
Viggiano *et al*. 2015[67], Italy	Low risk	Computer random number generator	Unclear risk	Method not stated	Low risk	ITT analysis not performed but reasons for attrition unlikely to have influenced outcomes, attrition bias analyses performed	Unclear risk	Not reported	Unclear risk	Insufficient information
Weeks *et al.*[22]	low risk	Random number generator	Low risk	Blind allocation at baseline	Low risk	ITT analysis performed	High risk	Researcher delivered program to intervention	Unclear risk	Insufficient information

First author, year, country, citation	Selection bias									
	Random sequence generation		Allocation concealment		Attrition bias		Detection bias		Reporting bias	
	Cochrane Judgment	Supporting evidence	Cochrane judgment	Supporting evidence	Cochrane judgment	Supporting evidence	Cochrane judgment	Supporting evidence	Cochrane judgment	Supporting evidence
2012 Australia								group and conducted measurements and analyses		

ITT, Intention to Treat;
