# Supplementary material for: Effectiveness of Lifestyle Interventions for Prevention of Harmful Weight Gain among Adolescents from Ethnic Minorities: A Systematic Review
Source: Int J Environ Res Public Health. 2020 Aug 20;17(17):6059. doi: 10.3390/ijerph17176059 (PMC7503574; doi:10.3390/ijerph17176059)
Supplement: Supplementary file 1 [file ijerph-17-06059-s001.zip › 1supplementary ijerph-870986/Table S1 Medline Search.pdf]

Table S1 Search Strategy Medline (Search 1)

#	Searches	Results
1	Adolescent/	1865754
2	Young Adult/	665066
3	students/	49191
4	adolescen*.tw.	206344
5	young adult*.tw.	69940
6	teen*.tw.	24337
7	youth*.tw.	51371
8	(young adj2 (person or people)).tw.	20650
9	highschool*.tw.	29
10	school*.tw.	217963
11	child*.tw.	1116151
12	or/1-11	3034740
13	BMI.tw.	98755
14	body mass index/	110217
15	weight gain/	28522
16	body weight/	179897
17	exp body weight changes/	63514
18	Body Mass Index.tw.	103598
19	Weight change*.tw.	8662
20	Weight loss*.tw.	65165
21	Anthropomet*.tw.	38627
22	obesity/	160493
23	or/13-22	546798
24	Primary prevention/	16754
25	(prevent* adj4 (weight or obes* or overweight)).tw.	12500
26	or/24-25	29042
27	health education/	57680
28	health promotion/	66492
29	Healthy People Programs/	1088
30	life style/	51773
31	healthy lifestyle/	664
32	sedentary lifestyle/	6804
33	Diet/	143191
34	Healthy Diet/	1444
35	Diet, western/	491
36	diet therapy/	10053
37	nutrition therapy/	1817
38	health promotion/	66492
39	sedentary lifestyle/	6804
40	exp SPORTS/	162783
41	exercise/	91854
42	swimming/	16320
43	exp RUNNING/	17879
44	exp WALKING/	46682
45	intervention*.tw.	695916

46	(Education adj4 (health or diet* or nutrition*)).tw.	46707
47	(Lifestyle adj4 (intervention* or change* or modif* or education or sedentary)).tw.	20423
48	(Life style adj4 (intervention* or change* or modif* or education or sedentary)).tw.	2049
49	(Diet* adj4 (intervention* or change* or modify* or educat* or therap* or program)).tw.	34935
50	(Nutrition adj4 (intervention* or change* or modify* or educat* or therap* or program)).tw.	12676
51	healthy eating*.tw.	4953
52	(weight adj3 (program* or management)).tw.	8054
53	Physical Activit*.tw.	76845
54	sport*.tw.	50590
55	exercise*.tw.	224388
56	or/27-55	1383450
57	12 and 23 and 26 and 56	3577
58	randomized controlled trial.pt.	461720
59	controlled clinical trial.pt.	92411
60	randomized.ab.	361455
61	placebo.ab.	173029
62	randomly.ab.	250125
63	trial.ab.	375176
64	groups.ab.	1562752
65	58 or 59 or 60 or 61 or 62 or 63 or 64	2284246
66	exp animals/ not humans.sh.	4462009
67	65 not 66	1881199
68	57 and 67	1352
69	limit 68 to (english language and yr="2005 -Current")	1172
