# Supplementary material for: Effectiveness of Lifestyle Interventions for Prevention of Harmful Weight Gain among Adolescents from Ethnic Minorities: A Systematic Review
Source: Int J Environ Res Public Health. 2020 Aug 20;17(17):6059. doi: 10.3390/ijerph17176059 (PMC7503574; doi:10.3390/ijerph17176059)
Supplement: Supplementary file 1 [file ijerph-17-06059-s001.zip › 1supplementary ijerph-870986/Table S2 Full Text Articles exluded with reasoning.pdf]

Table S2: Full-Text Articles excluded and supported reasoning (n=159)

**Abstract Only (n=24)**
1. Bohme P, Legrand K, Omorou AY, et al. A proportionate intervention in school led to improve overweight prevention without aggravating the health social inequalities. Obes Facts. 2016;1):34.
2. Briganti S, Zelaschi R, Ermetici F, et al. The Italian E.A.T. Project: Effectiveness of a multicomponent school-based health promotion study on measures of fatness and behavior in teenagers. Eating and Weight Disorders. 2014;19 (3):444-445.
3. Cunha D, Souza B, Pereira R, Sichieri R. Preventing excessive weight gain by encouraging healthy eating habits among adolescents in Brazil: A randomised community trial. FASEB Journal Conference: Experimental Biology. 2012;26(Meeting Abstracts).
4. Della Santa AP, Britz M, Deffeminis M, Dornell T, Martinez J, Cavalleri F. STUDY OF THE EFFECTIVENESS OF AN EDUCATIONAL MODEL TO PREVENT AND CONTROL CHILDHOOD OBESITY. Annals of Nutrition and Metabolism. 2013;63:928-928.
5. Della Torre SB, Dudley-Martin F, Kruseman M. Development and testing of an obesity prevention program for young children at risk, and their parents. Annals of Nutrition and Metabolism. 2011;58:260-260.
6. Emery CA, Richmond SA, Doyle-Baker PK. The efectivenes of a combined sport injury and obesity prevention program in junior high school. Paediatrics and Child Health. 2010;15 (SA):72A.
7. Ezendam N, Oenema A, Brug J. Short- and long-term effects of FATaintPHAT, a theory-based computer-tailored intervention to prevent unnecessary weight gain among adolescents. Psychol Health. 2010;25:31-32.
8. Hollis JL, Sutherland R, Campbell L, et al. A socio-ecologically framed, school-based physical activity intervention has beneficial effects on obesity outcomes in adolescents from low ses communities: The PA4E1 RCT. Obes Facts. 2015;1):110.
9. Jimenez EY, Sanders S, Vallabhan M, Kong AS. One year outcomes from an adolescent obesity prevention and management intervention in school-based health centers. J Adolesc Health. 2018;62 (2 Supplement 1):S120-S121.
10. Khatun M, Sonia NN, Mou MK, et al. Impact of nutrition education on overweight adolescent girls (10-19years) in the selected schools of Dhaka city. Annals of Nutrition and Metabolism. 2017;71 (Supplement 2):1254.
11. Low AK, Greening L, Harrell K, Fielder C, Roach J. The Efficacy of a Culturally Sensitive, Community/School-Based Childhood Obesity Prevention/Intervention Program. Obesity (Silver Spring). 2009;17:S209-S210.
12. Lubans D, Dewar D, Morgan P, et al. Two-year outcomes from the NEAT Girls obesity prevention cluster randomized controlled trial. Journal of Science and Medicine in Sport. 2013;1):e34.
13. Lubans D, Morgan P, Aguiar E, Callister R. Randomised controlled trial of the Physical Activity Leaders (PALs) program for low-active adolescent boys from disadvantaged secondary schools. Journal of Science and Medicine in Sport. 2010;1):e12.
14. Lubans D, Morgan P, Okely A, et al. Preventing obesity among adolescent girls: Outcomes of the nutrition and enjoyable activity for teen girls cluster randomized controlled trial. Journal of Science and Medicine in Sport. 2012;15 (SUPPL.1):S332.
15. Lubans D, Smith J, Morgan P, et al. Improving muscular fitness enhances psychological well-being in low-income adolescent boys: Findings from the ATLAS cluster RCT. Journal of Science and Medicine in Sport. 2015;1):e75.
16. Palcic JL, Johnston CA, Stansberry S, et al. Weight Change among Mexican American Students Involved in an Intensive Intervention to Prevent and Treat Obesity. FASEB Journal Conference: Experimental Biology. 2010;24(Meeting Abstracts).
17. Serge B, Emilie B, Nelly A, et al. Effectiveness of three overweight and obesity prevention strategies in high school adolescents. the pralimap controlled cluster randomised trial. Obes Rev. 2011;1):65-66.

18. Shneid Schuh DS, Pellanda LP, Ribas MR, et al. Healthy School, Happy School: Randomized clinical trial designed to stop obesity in children. European Heart Journal. 2016;37 (Supplement 1):550.<br>19. Simon C, Schweitzer B, Drai J, et al. Overweight prevention in adolescents by increasing physical activity: 6-year results of the randomized ICAPS study. Obes Rev. 2011;1):26.<br>20. Simon C, Schweitzer B, Oujaa M, et al. Overweight prevention in adolescents by increasing physical activity. Icaps, 4-year randomised controlled intervention. Int J Obes (Lond). 2008;32:S33-S33.<br>21. Smith J, Morgan P, Saunders K, Lubans D. Improving physical self-perception in adolescent boys from disadvantaged communities: Psychological outcomes from the PALs intervention. Journal of Science and Medicine in Sport. 2012;15 (SUPPL.1):S344-S345.<br>22. Tatrai-Nemeth K. Community approach of preventing childhood obesity: the first community based large-scale intervention pilot programme in Hungary based on the EPODE methodology. Annals of Nutrition and Metabolism. 2015;67:18-19.<br>23. Viggiano E, Viggiano A, Vicidomini C, et al. Kaledo, a new educational board-game for nutrition education: Cluster randomized trial of healthy lifestyle promotion. Obes Facts. 2012;1):260.<br>24. Xu F, Wang Z, Ye Q, et al. Policy-oriented, school-based physical activity intervention to prevent childhood obesity in China (the health legacy project of the second Summer Youth Olympic Games): A cluster randomised trial. The Lancet Diabetes and Endocrinology. 2016;ISSUE 3):S9.
**Dissertation (n=4)**
1. Bauer KW. The family environment and adolescent girls' weight and weight-related behaviors: Implications for obesity prevention programs. Dissertation Abstracts International: Section B: The Sciences and Engineering. 2011;71(7-B):4205.<br>2. Foster LK. Examining the physical and mental health effects of an obesity prevention program in high risk adolescent females: A pilot study. Dissertation Abstracts International Section A: Humanities and Social Sciences. 2015;75(7-A(E)):No Pagination Specified.<br>3. Ritchie T. Evaluation of the Impact of the Creating Opportunity for Personal Empowerment (COPE) Healthy Lifestyles Thinking, Emotions, Exercise, and Nutrition (TEEN) Program in a Rural High School Health Class, West Virginia University; 2011.<br>4. Setlow PD. Evaluation of the effectiveness of healthy me a nutritional and exercise program for African American adolescents in an urban school utilizing the cope/teen program. Dissertation Abstracts International: Section B: The Sciences and Engineering. 2014;75(3-B(E)):No Pagination Specified.
**Duplicate (n=6)**
1. Black MM, Hager ER, Le K, et al. Challenge!: A Mentorship Model of Health Promotion and Obesity Prevention Among Urban, African American Adolescents. Pediatrics. 2010;126(2):280-288.<br>2. Lubans DR, Morgan PJ, Okely AD, et al. Preventing Obesity Among Adolescent Girls. Arch Pediatr Adolesc Med. 2012;166(9):821-827.<br>3. Omorou AY, Langlois J, Lecomte E, Vuillemin A, Briancon S, Group PT. Adolescents' Physical Activity and Sedentary Behavior: A Pathway in Reducing Overweight and Obesity. The PRALIMAP 2-Year Cluster Randomized Controlled Trial. J Phys Act Health. 2015;12(5):628-635.<br>4. Whittemore R, Grey M, Jeon S. Internet obesity prevention programs are effective for adolescents. Diabetes. 2012;1):A210.<br>1. Dunker KLL, Claudino AM. Preventing weight-related problems among adolescent girls: A cluster randomized trial comparing the Brazilian 'New Moves' program versus observation. Obesity Research & Clinical Practice. 2018;12(1):102-115.<br>2. Viggiano E, Viggiano A, Di Costanzo A, et al. Healthy lifestyle promotion in primary schools through the board game Kaledo: a pilot cluster randomized trial. Eur J Pediatr. 2018;177(9):1371-1375.
**Not relevant outcome (n=13)**
1. Abood DA, Black DR, Coster DC. Evaluation of a school-based teen obesity prevention minimal intervention. J Nutr Educ Behav. 2008;40(3):168-174.<br>2. Cotts TB, Durussel-Weston JE, Goldberg CS, Palma-Davis LM, Aaronson SM, Eagle KA. Project healthy schools: a school based health education program to prevent childhood obesity. Agro Food Ind Hi-Tech. 2007;18(5):32-34.

3. Durrer D, Schutz Y. ADOS: an educational primary prevention programme for preventing excess body weight in adolescents. Int J Obes (Lond). 2008;32:S72-S76.
4. Lubans D, Syjva K. Controlled evaluation of a physical activity intervention for senior school students: Effects of the lifetime activity program. J Sport Exerc Psychol. 2006;28(3):252-268.
5. Mauriello LM, Driskell MM, Sherman KJ, Johnson SS, Prochaska JM, Prochaska JO. Acceptability of a school-based intervention for the prevention of adolescent obesity. J Sch Nurs. 2006;22(5):269-277.
6. Ochoa-Aviles A, Verstraeten R, Huybregts L, et al. A school-based intervention improved dietary intake outcomes and reduced waist circumference in adolescents: a cluster randomized controlled trial. Nutr J. 2017;16:12.
7. Omorou AY, Langlois J, Lecomte E, Vuillemin A, Briancon S. Erratum: Adolescents' Physical Activity and Sedentary Behavior: A Pathway in Reducing Overweight and Obesity. The PRALIMAP 2-Year Cluster Randomized Controlled Trial (Journal of physical activity & health). J Phys Act Health. 2015;12(5):628-635.
8. Siegrist M, Hanssen H, Lammel C, Haller B, Halle M. A cluster randomised school-based lifestyle intervention programme for the prevention of childhood obesity and related early cardiovascular disease (JuvenTUM 3). BMC Public Health. 2011;11:258.
9. Vargas ICD, Sichieri R, Sandre-Pereira G, da Veiga GV. Evaluation of an obesity prevention program in adolescents of public schools. Rev Saude Publica. 2011;45(1):59-68.
1. Byrne JLS, Wild TC, Maximova K, et al. A brief eHealth tool delivered in primary care to help parents prevent childhood obesity: a randomized controlled trial. Pediatr Obes. 2018;13(11):659-667.
2. Hager ER, Song HJ, Lane HG, Guo HH, Jaspers LH, Lopes MA. Pilot-Testing an Intervention to Enhance Wellness Policy Implementation in Schools: Wellness Champions for Change. Journal of Nutrition Education and Behavior. 2018;50(8):765-775.
3. Saez L, Langlois J, Legrand K, et al. Reach and Acceptability of a Mobile Reminder Strategy and Facebook Group Intervention for Weight Management in Less Advantaged Adolescents: Insights From the PRALIMAP-INES Trial. Jmir Mhealth and Uhealth. 2018;6(5).
4. Trude ACB, Kharmats AY, Jones-Smith JC, Gittelsohn J. Exposure to a multi-level multi-component childhood obesity prevention community- randomized controlled trial: patterns, determinants, and implications. Trials. 2018;19.

Not in English (n=10)

1. Eichhorn C, Bodner L, Liebl S, et al. BEO'S - Physical Activity and Healthy Eating at Schools in Oberfranken, Bavaria Concept and First Results of a Resource-Oriented, Systemic Approach in School-Based Health Promotion. Gesundheitswesen. 2012;74(2):104-111.
2. Grao-Cruces A, Nuviala A, Fernandez-Martinez A. Assessment of the Sport Schools program: Body composition, physical activity and cardiorespiratory fitness in adolescents. Retos. 2015(27):105-108.
3. Honicky M, Kuhl AM, Melhem ARD. Nutritional intervention in overweight children and adolescents. RBONE-Rev Bras Obes Nutr Emagrecimento. 2017;11(66):486-495.
4. Kain J, Leyton B, Concha F, Salazar G, Lobos L, Vio F. Effect of counselling school teachers on healthy lifestyle on the impact of a program to reduce childhood obesity. Rev Medica Chile. 2010;138(2):181-187.
5. Kain J, Uauy R, Leyton B, Cerda R, Olivares S, Vio F. Effectiveness of a dietary and physical activity intervention to prevent obesity in school age children. Rev Medica Chile. 2008;136(1):22-30.
6. Kim YH, Yang YO. Effects of walking exercise on metabolic syndrome risk factors and body composition in obese middle school girls. Taehan kanho hakhoe chi. 2005;35(5):858-867.
7. Plachta-Danielzik S, Landsberg B, Lange D, Langnase K, Muller MJ. 15 years of the Kiel Obesity Prevention Study (KOPS). Results and its importance for obesity prevention in children and adolescents. Bundesgesundheitsblatt-Gesund. 2011;54(3):304-312.
8. Poppelmeyer C, Helk O, Mehany S, Hauer R, Pachinger O, Widhalm K. The Viennese Prevention Study (EDDY): initial results. Padiatr Padol. 2016;51(3):104-108.
9. Quizan-Plata T, Meneses LV, Romero JE, Villar AVB, Zavala RGD. EDUCATIONAL PROGRAM HAD A POSITIVE EFFECT ON THE INTAKE OF FAT, FRUITS AND VEGETABLES AND PHYSICAL

ACTIVITY IN STUDENTS ATTENDING PUBLIC ELEMENTARY SCHOOLS OF MEXICO. Nutr Hosp. 2014;30(3):552-561.<br>10. Schraffl M, Helk O, Winkler A, et al. EDDY - Effect of sport and diet training to prevent obesity and secondary diseases and to influence young children's lifestyle: An innovative Prevention Project in Vienna. Wien Klin Wochen. 2012;124(17-18):663-663.
**Not relevant population (n=52)**
1. Branscum P, Sharma M, Leigh Wang L, Wilson BRA, Rojas-Guyler L. A true challenge for any superhero: An evaluation of a comic book obesity prevention program. Family and Community Health. 2013;36(1):63-76.<br>2. Bruss MB, Michael TJ, Morris JR, et al. Childhood obesity prevention: an intervention targeting primary caregivers of school children. Obesity (Silver Spring). 2010;18(1):99-107.<br>3. Byrd-Bredbenner C, Worobey J, Martin-Biggers J, Hongu N, Hernandez G. HomeStyles: Shaping Home Environments and Lifestyle Practices to Prevent Childhood Obesity: A Randomized Controlled Trial. J Nutr Educ Behav. 2015;47(4):S99-S99.<br>4. Carrel AL, Clark RR, Peterson SE, Nemeth BA, Sullivan J, Allen DB. Improvement of fitness, body composition, and insulin sensitivity in overweight children in a school-based exercise program - A randomized, controlled study. Arch Pediatr Adolesc Med. 2005;159(10):963-968.<br>5. Cebolla i Marti A, Alvarez-Pitti JC, Guixeres Provinciale J, Lison JF, Banos Rivera R. Alternative options for prescribing physical activity among obese children and adolescents: brisk walking supported by an exergaming platform. Nutr Hosp. 2014;31(2):841-848.<br>6. Christison A, Khan HA. Exergaming for Health: A Community-Based Pediatric Weight Management Program Using Active Video Gaming. Clin Pediatr (Phila). 2012;51(4):382-388.<br>7. Cullen KW, Smalling AL, Thompson D, Watson KB, Reed D, Konzelmann K. Creating Healthful Home Food Environments: Results of a Study with Participants in the Expanded Food and Nutrition Education Program. Journal of Nutrition Education and Behavior. 2009;41(6):380-388.<br>8. Cunha DB, de Souza Bda S, Pereira RA, Sichieri R. Effectiveness of a randomized school-based intervention involving families and teachers to prevent excessive weight gain among adolescents in Brazil. PLoS ONE. 2013;8(2):e57498.<br>9. da Silva LS, Fisberg M, de Souza Pires MM, Nassar SM, Sottovia CB. The effectiveness of a physical activity and nutrition education program in the prevention of overweight in schoolchildren in Criciuma, Brazil. Eur J Clin Nutr. 2013;67(11):1200-1204.<br>10. Damon S, Dietrich S, Widhalm K. PRESTO - Prevention Study of Obesity: A project to prevent obesity during childhood and adolescence. Acta Paediatr. 2005;94:47-48.<br>11. Davison KK, Jurkowski JM, Li K, Kranz S, Lawson HA. A childhood obesity intervention developed by families for families: results from a pilot study. Int. 2013;10:3.<br>12. Donnelly JE, Sullivan DK, Smith BK, et al. Alteration of dietary fat intake to prevent weight gain: Jayhawk observed eating trial. Obesity (Silver Spring). 2008;16(1):107-112.<br>13. Gallotta MC, Iazzoni S, Emerenziani GP, et al. Effects of combined physical education and nutritional programs on schoolchildren's healthy habits. Peerj. 2016;4:20.<br>14. Gentile DA, Welk G, Eisenmann JC, et al. Evaluation of a multiple ecological level child obesity prevention program: Switch what you Do, View, and Chew. BMC Med. 2009;7:49.<br>15. Grydeland M, Bergh IH, Bjelland M, et al. Intervention effects on physical activity: the HEIA study - a cluster randomized controlled trial. International Journal of Behavioral Nutrition and Physical Activity. 2013;10:13.<br>16. Gutin B, Yin Z, Johnson M, Barbeau P. Preliminary findings of the effect of a 3-year after-school physical activity intervention on fitness and body fat: The Medical College of Georgia Fitkid Project. Int J Pediatr Obes. 2008;3(SUPPL.1):3-9.<br>17. Hollar D, Messiah S, Hollar TL, Lopez-Mitnik G, Agatston AS. School-Based Obesity and Related Cardiovascular Disease Prevention Intervention Effect on Weight and Academic Performance: Three Year Results. Journal of the American College of Cardiology. 2009;53(10):A399-A399.

18. Hollar D, Messiah S, Lopez-Mitnik G, Hollar T, Almon M, Agatston A. Effect of an Elementary School-Based Obesity Prevention Intervention on Weight and Academic Performance among Low Income Children. Obesity (Silver Spring). 2008;16:S57-S57.
19. Hollar D, Zhou W, Riggle Z. Afterschool soccer fitness and nutrition program improves BMI percentile, waist circumference, and fitness levels in participants compared to nonparticipants. Circulation Conference: American Heart Association's Epidemiology and Prevention/Lifestyle and Cardiometabolic Health. 2015;131(SUPPL. 1).
20. Johnston CA, Palcic J, George CS, El-Mubasher AA, Tyler C, Foreyt JP. Weight change among Mexican American students involved in an intensive intervention to prevent and treat obesity: 5-year results. Obesity (Silver Spring). 2011;1):S111-S112.
21. Jones M, Luce KH, Osborne MI, et al. Randomized, controlled trial of an internet-facilitated intervention for reducing binge eating and overweight in adolescents. Pediatrics. 2008;121(3):453-462.
22. Jones RA, Kelly J, Cliff DP, Batterham M, Okely AD. Acceptability and Potential Efficacy of Single-Sex After-School Activity Programs for Overweight and At-Risk Children: The Wollongong SPORT RCT. Pediatr Exerc Sci. 2015;27(4):535-545.
23. King MH, Lederer AM, Sovinski D, et al. Implementation and evaluation of the HEROES Initiative: A tri-state coordinated school health program to reduce childhood obesity. Health Promot Pract. 2014;15(3):395-405.
24. Klakk H, Chinapaw M, Heidemann M, Andersen LB, Wedderkopp N. Effect of four additional physical education lessons on body composition in children aged 8-13 years - a prospective study during two school years. BMC Pediatr. 2013;13:8.
25. Leidy HJ, Hoertel HA, Douglas SM, Higgins KA, Shafer RS. A high-protein breakfast prevents body fat gain, through reductions in daily intake and hunger, in "Breakfast skipping" adolescents. Obesity (Silver Spring, Md). 2015;23(9):1761-1764.
26. Lemon SC, Wang ML, Wedick NM, et al. Weight gain prevention in the school worksite setting: Results of a multi-level cluster randomized trial. Prev Med. 2014;60:41-47.
27. Lim H, Kim J, Wang YF, Min J, Carvajal NA, Lloyd CW. Child health promotion program in South Korea in collaboration with US National Aeronautics and Space Administration: Improvement in dietary and nutrition knowledge of young children. Nutr Res Pract. 2016;10(5):555-562.
28. Liu AL, Hu XQ, Ma GS, et al. Report on childhood obesity in China (6) Evaluation of a classroom-based physical activity promotion program. Biomedical and Environmental Sciences. 2007;20(1):19-23.
29. Love-Osborne K, Fortune R, Sheeder J, Federico S, Haemer MA. School-based health center-based treatment for obese adolescents: feasibility and body mass index effects. Child. 2014;10(5):424-431.
30. Markert J, Herget S, Petroff D, et al. Telephone-based adiposity prevention for families with overweight children (T.A.F.F.-Study): one year outcome of a randomized, controlled trial. Int J Environ Res Public Health. 2014;11(10):10327-10344.
31. Meucci M, Cook C, Curry CD, Guidetti L, Baldari C, Collier SR. Effects of supervised exercise program on metabolic function in overweight adolescents. World J Pediatr. 2013;9(4):307-311.
32. Neelon SE, Brouwer RJ, Ostbye T, et al. A community-based intervention increases physical activity and reduces obesity in school-age children in North Carolina. Child. 2015;11(3):297-303.
33. Olvera N, Scherer R, McLeod J, et al. BOUNCE: An Exploratory Healthy Lifestyle Summer Intervention for Girls. Am J Health Behav. 2010;34(2):144-155.
34. Parra-Medina D, Mojica C, Liang Y, Ouyang Y, Ramos AI, Gomez I. Promoting Weight Maintenance among Overweight and Obese Hispanic Children in a Rural Practice. Child. 2015;11(4):355-363.
35. Plachta-Danielzik S, Landsberg B, Lange D, Seiberl J, Muller MJ. Eight-year follow-up of school-based intervention on childhood overweight--the Kiel Obesity Prevention Study. Obes Facts. 2011;4(1):35-43.
36. Ranucci C, Pippi R, Buratta L, et al. Effects of an Intensive Lifestyle Intervention to Treat Overweight/Obese Children and Adolescents. Biomed Res Int. 2017:11.

37. Rerksuppaphol L, Rerksuppaphol S. Internet based obesity prevention program for Thai school children-a randomized control trial. Journal of Clinical and Diagnostic Research. 2017;11(3):SC07-SC11.
38. Sadeghi B, Kaiser LL, Schaefer S, et al. Multifaceted community-based intervention reduces rate of BMI growth in obese Mexican-origin boys. Pediatr Obes. 2017;12(3):247-256.
39. Salameh AB, Al-sheyab N, El-hneiti M, Shaheen A, Williams LM, Gallagher R. Effectiveness of a 12-week school-based educational preventive programme on weight and fasting blood glucose in "at-risk" adolescents of type 2 diabetes mellitus: Randomized controlled trial. International Journal of Nursing Practice. 2017;23(3):8.
40. Simon C, Kellou N, Dugas J, et al. A socio-ecological approach promoting physical activity and limiting sedentary behavior in adolescence showed weight benefits maintained 2.5 years after intervention cessation. Int J Obes (Lond). 2014;38(7):936-943.
41. Simon C, Schweitzer B, Oujaa M, et al. "Successful overweight prevention in adolescents by increasing physical activity: A 4-year randomized controlled intervention": Corrigendum. Int J Obes (Lond). 2008;32(10):1606.
42. Stice E, Rohde P, Shaw H, Marti C. Efficacy trial of a selective prevention program targeting both eating disorders and obesity among female college students: 1- and 2-year follow-up effects. Journal of Consulting and Clinical Psychology. 2013;81(1):183-189.
43. Stice E, Rohde P, Shaw H, Marti CN. Efficacy Trial of a Selective Prevention Program Targeting Both Eating Disorder Symptoms and Unhealthy Weight Gain Among Female College Students. Journal of Consulting and Clinical Psychology. 2012;80(1):164-170.
44. Taylor RW, McAuley KA, Barbezat W, Farmer VL, Williams SM, Mann JI. Two-year follow-up of an obesity prevention initiative in children: the APPLE project. Am J Clin Nutr. 2008;88(5):1371-1377.
45. Weintraub DL, Tirumalai EC, Haydel KF, Fujimoto M, Fulton JE, Robinson TN. Team sports for overweight children: the Stanford Sports to Prevent Obesity Randomized Trial (SPORT). Arch Pediatr Adolesc Med. 2008;162(3):232-237.
46. Wong PC, Chia MY, Tsou IY, et al. Effects of a 12-week exercise training programme on aerobic fitness, body composition, blood lipids and C-reactive protein in adolescents with obesity. Ann Acad Med Singapore. 2008;37(4):286-293.
47. Xu HQ, Li YP, Zhang Q, et al. Comprehensive school-based intervention to control overweight and obesity in China: a cluster randomized controlled trial. Asia Pac J Clin Nutr. 2017;26(6):1139-1151.
1. Hovell MF, Schmitz KE, Liles S, et al. A randomized controlled trial of orthodontist-based brief advice to prevent child obesity. Contemporary Clinical Trials. 2018;70:53-61.
2. Ickovics JR, Duffany KO, Shebl FM, et al. Implementing School-Based Policies to Prevent Obesity: Cluster Randomized Trial. Am J Prev Med. 2019;56(1):E1-E11.
3. Wang Z, Xu F, Ye Q, et al. Childhood obesity prevention through a community-based cluster randomized controlled physical activity intervention among schools in china: the health legacy project of the 2nd world summer youth olympic Games (YOG-Obesity study). International Journal of Obesity. 2018;42(4):625-633.
4. Wong AX, Sanchez-Gonzalez MA, Son WM, Kwak YS, Park SY. The Effects of a 12-Week Combined Exercise Training Program on Arterial Stiffness, Vasoactive Substances, Inflammatory Markers, Metabolic Profile, and Body Composition in Obese Adolescent Girls. Pediatric Exercise Science. 2018;30(4):480-486.
5. Bagherniya M, Mostafavi Darani F, Sharma M, et al. Assessment of the Efficacy of Physical Activity Level and Lifestyle Behavior Interventions Applying Social Cognitive Theory for Overweight and Obese Girl Adolescents. J Res Health Sci. 2018;18(2):e00409.

Not a RCT (n=34)

1. Anguita M. Effective weight management to prevent obesity. Independent Nurse. 2010:26-27.
2. Ardic A, Erdogan S. The effectiveness of the COPE healthy lifestyles TEEN program: a school-based intervention in middle school adolescents with 12-month follow-up. J Adv Nurs. 2017;73(6):1377-1389.

3. Barry CL, Gollust SE, McGinty EE, Niederdeppe J. Effects of messages from a media campaign to increase public awareness of childhood obesity. Obesity (Silver Spring). 2014;22(2):466-473.
4. Borys JM, Roillet M, Beysens J, Jacobs N, Neve J. CHILDHOOD OBESITY PREVENTION : A SIGNIFICANT DECREASE OF OVERWEIGHT PREVALENCE IN TWO PILOT TOWNS OF THE VIASANO PROGRAMME AFTER 2 YEARS OF INTERVENTION. Annals of Nutrition and Metabolism. 2013;63:919-919.
5. Burguera B, Colom A, Pinero E, et al. ACTYBOSS: Activity, Behavioral Therapy in Young Subjects - After-School Intervention Pilot Project on Obesity Prevention. Obes Facts. 2011;4(5):400-406.
6. Castro DC, Samuels M, Harman AE. Growing Healthy Kids: A community garden-based obesity prevention program. Am J Prev Med. 2013;44(3, Suppl 3):S193-S199.
7. Craven KW, Moore JB, Swart AS, Keene AF, Kolasa KM. School-Based Nutrition Education Intervention: Effect on Achieving a Healthy Weight Among Overweight Ninth-Grade Students. J Public Health Manag Pract. 2011;17(2):141-146.
8. Elinder LS, Heinemans N, Hagberg J, Quetel A-K, Hagstromer M. A participatory and capacity-building approach to healthy eating and physical activity-SCIP-school: A 2-year controlled trial. The International Journal of Behavioral Nutrition and Physical Activity Vol 9 2012, ArtID 145. 2012;9.
9. Evans RK, Franco RL, Stern M, et al. Evaluation of a 6-month multi-disciplinary healthy weight management program targeting urban, overweight adolescents: Effects on physical fitness, physical activity, and blood lipid profiles. Int J Pediatr Obes. 2009;4(3):130-133.
10. Flattum C, Friend S, Neumark-Sztainer D, Story M. Motivational Interviewing as a Component of a School-Based Obesity Prevention Program for Adolescent Girls. J Am Diet Assoc. 2009;109(1):91-94.
11. Fotu KF, Millar L, Mavoa H, et al. Outcome results for the Ma'alahi Youth Project, a Tongan community-based obesity prevention programme for adolescents. Obes Rev. 2011;12 Suppl 2:41-50.
12. Gatti C, Suhas E, Cote S, Anassour Laouan-Sidi E, Dewailly E, Lucas M. Obesity and metabolic parameters in adolescents: A school-based intervention program in French Polynesia. J Adolesc Health. 2015;56(2):174-180.
13. Green DJ, Cable T, Reilly JJ, Paton JY, McColl JH, Williamson A. Physical activity to prevent obesity in young children: BMI in the BMJ...Reilly JJ, Kelly L, Montgomery C, Williamson A, Fisher A, McColl JH, et al. Physical activity to prevent obesity in young children: cluster randomised controlled trial. BMJ 2006; 333:1041-3 (18 November). BMJ: British Medical Journal (International Edition). 2006;333(7579):1171-1172.
14. Kremer P, Waqa G, Vanualailai N, et al. Reducing unhealthy weight gain in Fijian adolescents: results of the Healthy Youth Healthy Communities study. Obes Rev. 2011;12 Suppl 2:29-40.
15. Lazorick S, Crawford Y, Gilbird A, et al. Long-term obesity prevention and the Motivating Adolescents with Technology to CHOOSE HealthTM program. Child. 2014;10(1):25-33.
16. Lazorick S, Fang XM, Crawford Y. The MATCH Program: Long-Term Obesity Prevention Through a Middle School Based Intervention. Child. 2016;12(2):103-112.
17. Maatoug J, Msakni Z, Zammit N, et al. School-based intervention as a component of a comprehensive community program for overweight and obesity prevention, Sousse, Tunisia, 2009-2014. Preventing Chronic Disease: Public Health Research, Practice, and Policy Vol 12 2015, ArtID 140518. 2015;12.
18. Malakellis M, Hoare E, Sanigorski A, et al. School-based systems change for obesity prevention in adolescents: outcomes of the Australian Capital Territory 'It's Your Move!'. Aust N Z J Public Health. 2017;41(5):490-496.
19. Millar L, Kremer P, de Silva-Sanigorski A, et al. Reduction in overweight and obesity from a 3-year community-based intervention in Australia: the 'It's Your Move!' project. Obes Rev. 2011;12:20-28.
20. Parra-Medina D, Liang YY, Yin ZN, Esparza L, Lopez L. Weight Outcomes of Latino Adults and Children Participating in the Y Living Program, a Family-Focused Lifestyle Intervention, San Antonio, 2012-2013. Prev Chronic Dis. 2015;12:13.

21. Peterson KE, Spadano-Gasbarro JL, Greaney ML, et al. Three-Year Improvements in Weight Status and Weight-Related Behaviors in Middle School Students: The Healthy Choices Study. PLoS ONE. 2015;10(8):12.
22. Pettman T, Magarey A, Mastersson N, Wilson A, Dollman J. Improving weight status in childhood: results from the eat well be active community programs. Int J Public Health. 2014;59(1):43-50.
23. Polacsek M, Orr J, O'Brien LM, Rogers VW, Fanburg J, Gortmaker SL. Sustainability of key Maine Youth Overweight Collaborative improvements: a follow-up study. Child. 2014;10(4):326-333.
24. Pope M. Preventing Weight Gain in Children Who Are School Age and African-American. Pediatr. 2016;28(2):207-216.
25. Robbins LB, Pfeiffer KA, Maier KS, Lo YJ, Wesolek SM. Pilot Intervention to Increase Physical Activity Among Sedentary Urban Middle School Girls: A Two-Group Pretest-Posttest Quasi-Experimental Design. J Sch Nurs. 2012;28(4):302-315.
26. Salimin N, Elumalai G, Shahril MI, Subramaniam G. The Effectiveness of 8 Weeks Physical Activity Program among Obese Students. Amsterdam: Elsevier Science Bv; 2015.
27. Schwarte L, Samuels SE, Capitman J, Ruwe M, Boyle M, Flores G. The Central California Regional Obesity Prevention Program: Changing Nutrition and Physical Activity Environments in California's Heartland. Am J Public Health. 2010;100(11):2124-2128.
28. Spiliotis BE. Community intervention programs for the prevention and treatment of obesity in the child and adult. Int J Obes (Lond). 2008;32:S13-S13.
29. Yang Y, Kang B, Lee E, et al. Effect of an obesity prevention program focused on motivating environments in childhood: A school-based prospective study. Int J Obes (Lond). 2017;41(7):1027-1034.
30. Zhang YX, Zhou JY, Zhao JS, Chu ZH. The role of 1-h physical activity every day in preventing obesity in adolescents in Shandong, China. Eur J Pediatr. 2013;172(3):325-330.
1. Meng Y, Manore MM, Schuna JM, Jr., Patton-Lopez MM, Branscum A, Wong SS. Promoting Healthy Diet, Physical Activity, and Life-Skills in High School Athletes: Results from the WAVE Ripples for Change Childhood Obesity Prevention Two-Year Intervention. Nutrients. 2018;10(7):23.
2. Meng Y, Wong SS, Manore MM, Patton-Lopez M. WAVE~Ripples for Change Obesity Two-Year Intervention in High School Soccer Players: Process Evaluation, Best Practices, and Youth Engagement. Nutrients. 2018;10(6):01.
3. Meng Y, Wong SS, Manore MM, Patton-Lopez M. WAVE similar to Ripples for Change Obesity Two-Year Intervention in High School Soccer Players: Process Evaluation, Best Practices, and Youth Engagement. Nutrients. 2018;10(6).
4. Partridge S, Singleton A, Cohen P, et al. Development of a Bank of Text Messages Targeting Healthy Eating, Physical Activity, and Social Wellbeing In Adolescents At Risk of Obesity. Global heart. 2018;Conference: world congress of cardiology & cardiovascular health. 2018. United arab emirates 13(4):493.

Not relevant intervention (n=12)

1. Chen JL, Guedes CM, Cooper BA, Lung AE. Short-Term Efficacy of an Innovative Mobile Phone Technology-Based Intervention for Weight Management for Overweight and Obese Adolescents: Pilot Study. Interact J Med Res. 2017;6(2):12.
2. Correa NP, Murray NG, Mei CA, et al. CAN DO Houston: A Community-Based Approach to Preventing Childhood Obesity. Prev Chronic Dis. 2010;7(4):11.
3. Doyle AC, Goldschmidt A, Huang C, Winzelberg AJ, Taylor CB, Wilfley DE. Reduction of overweight and eating disorder symptoms via the Internet in adolescents: a randomized controlled trial. J Adolesc Health. 2008;43(2):172-179.
4. Luce KH, Osborne MI, Winzelberg AJ, et al. Application of an Algorithm-Driven Protocol to Simultaneously Provide Universal and Targeted Prevention Programs. Int J Eat Disord. 2005;37(3):220-226.
5. Ranucci C, Pippi R, Buratta L, et al. Effects of an Intensive Lifestyle Intervention to Treat Overweight/Obese Children and Adolescents. Biomed Res Int. 2017:11.

6. Sutherland R, Reeves P, Campbell E, et al. Cost effectiveness of a multi-component school-based physical activity intervention targeting adolescents: the 'Physical Activity 4 Everyone' cluster randomized trial. Int. 2016;13:94.
7. Tanofsky-Kraff M, Shomaker LB, Wilfley DE, et al. Excess weight gain prevention in adolescents: Three-year outcome following a randomized controlled trial. J Consult Clin Psychol. 2017;85(3):218-227.
8. Tanofsky-Kraff M, Shomaker LB, Wilfley DE, et al. Targeted prevention of excess weight gain and eating disorders in high-risk adolescent girls: A randomized controlled trial. Am J Clin Nutr. 2014;100(4):1010-1018.
9. Tanofsky-Kraff M, Wilfley DE, Young JF, et al. A pilot study of interpersonal psychotherapy for preventing excess weight gain in adolescent girls at-risk for obesity. Int J Eat Disord. 2010;43(8):701-706.
1. Freira S, Lemos MS, Fonseca H, et al. Anthropometric outcomes of a motivational interviewing school-based randomized trial involving adolescents with overweight. Eur J Pediatr. 2018;177(7):1121-1130.
2. Nct. Project Health: enhancing Effectiveness of a Dissonance-Based Obesity Prevention Program. Https://clinicaltrialsgov/show/nct03710746. 2018.
3. Yang Q, Huang G, Tian Q, et al. "Living High-Training Low" improved weight loss and glucagon-like peptide-1 level in a 4-week weight loss program in adolescents with obesity: A pilot study. Medicine (Baltimore). 2018;97(8):e9943.

Protocol Only (n=4)

1. Ezendam NP, Oenema A, van de Looij-Jansen PM, Brug J. Design and evaluation protocol of "FATaintPHAT", a computer-tailored intervention to prevent excessive weight gain in adolescents. BMC Public Health. 2007;7:324.
2. Li Y, Hu X, Zhang Q, et al. The nutrition-based comprehensive intervention study on childhood obesity in China (NISCOC): a randomised cluster controlled trial. BMC Public Health. 2010;10:229.
3. Robbins LB, Pfeiffer KA, Vermeesch A, et al. "Girls on the Move" intervention protocol for increasing physical activity among low-active underserved urban girls: a group randomized trial. BMC Public Health. 2013;13:12.
4. Simons M, Chinapaw MJ, van de Bovenkamp M, et al. Active video games as a tool to prevent excessive weight gain in adolescents: rationale, design and methods of a randomized controlled trial. BMC Public Health. 2014;14:275.
